# Sex differences in clinical phenotypes of behavioral variant frontotemporal dementia

**DOI:** 10.1002/alz.14608

**Published:** 2025-04-25

**Authors:** Xulin Liu, Sterre C. M. de Boer, Kasey Cortez, Jackie M. Poos, Ignacio Illán‐Gala, Hilary Heuer, Leah K. Forsberg, Kaitlin Casaletto, Molly Memel, Brian S. Appleby, Sami Barmada, Andrea Bozoki, David Clark, Yann Cobigo, Ryan Darby, Bradford C. Dickerson, Kimiko Domoto‐Reilly, Douglas R. Galasko, Daniel H. Geschwind, Nupur Ghoshal, Neill R. Graff‐Radford, Ian M. Grant, Ging‐Yuek Robin Hsiung, Lawrence S. Honig, Edward D. Huey, David Irwin, Kejal Kantarci, Gabriel C. Léger, Irene Litvan, Ian R. Mackenzie, Joseph C. Masdeu, Mario F. Mendez, Chiadi U. Onyike, Belen Pascual, Peter Pressman, Ece Bayram, Eliana Marisa Ramos, Erik D. Roberson, Emily Rogalski, Arabella Bouzigues, Lucy L. Russell, Phoebe H. Foster, Eve Ferry‐Bolder, Mario Masellis, John van Swieten, Lize Jiskoot, Harro Seelaar, Raquel Sanchez‐Valle, Robert Laforce, Caroline Graff, Daniela Galimberti, Rik Vandenberghe, Alexandre de Mendonça, Pietro Tiraboschi, Isabel Santana, Alexander Gerhard, Johannes Levin, Sandro Sorbi, Markus Otto, Florence Pasquier, Simon Ducharme, Chris R. Butler, Isabelle Le Ber, Elizabeth Finger, James B. Rowe, Matthis Synofzik, Fermin Moreno, Barbara Borroni, Brad F. Boeve, Adam L. Boxer, Howie J. Rosen, Yolande A. L. Pijnenburg, Jonathan D. Rohrer, Maria Carmela Tartaglia, the ALLFTD Consortium and the GENFI Consortium

**Affiliations:** ^1^ Krembil Research Institute University Health Network Toronto Canada; ^2^ Tanz Centre for Research in Neurodegenerative Diseases University of Toronto Toronto Canada; ^3^ Alzheimer Center Amsterdam Neurology Vrije Universiteit Amsterdam Amsterdam UMC location VUmc Amsterdam Amsterdam the Netherlands; ^4^ Amsterdam Neuroscience, Neurodegeneration Amsterdam the Netherlands; ^5^ The University of Sydney School of Psychology and Brain & Mind Centre Sydney Australia; ^6^ Department of Neurology and Alzheimer Center Erasmus MC Erasmus MC University Medical Center Rotterdam the Netherlands; ^7^ Sant Pau Memory Unit Department of Neurology Biomedical Research Institute Sant Pau Hospital de la Santa Creu i Sant Pau Universitat Autònoma de Barcelona Hospital de la Santa Creu i Sant Pau Barcelona Spain; ^8^ Memory and Aging Center Department of Neurology Weill Institute for Neurosciences University of California Sandler Neurosciences Center, San Francisco San Francisco USA; ^9^ Department of Neurology Mayo Clinic Rochester USA; ^10^ Department of Neurology Case Western Reserve University Cleveland USA; ^11^ University of Michigan Ann Arbor USA; ^12^ University of North Carolina Chapel Hill USA; ^13^ Indiana University Indianapolis USA; ^14^ Vanderbilt University Nashville USA; ^15^ Department of Neurology Massachusetts General Hospital and Harvard Medical School Boston USA; ^16^ Department of Neurology University of Washington Seattle USA; ^17^ University of California San Diego La Jolla USA; ^18^ Department of Neurology David Geffen School of Medicine University of California Los Angeles USA; ^19^ Departments of Neurology and Psychiatry Washington University School of Medicine in St Louis St. Louis USA; ^20^ Mayo Clinic Jacksonville USA; ^21^ Department of Psychiatry and Behavioral Sciences Mesulam Center for Cognitive Neurology and Alzheimer's Disease Northwestern Feinberg School of Medicine Chicago USA; ^22^ University of British Columbia 2329 West Mall Vancouver Canada; ^23^ Neurology Department and Taub Institute Columbia University Irving Medical Center New York USA; ^24^ Department of Psychiatry and Human Behavior Alpert Medical School of Brown University Providence USA; ^25^ Perelman School of Medicine University of Pennsylvania Philadelphia USA; ^26^ Department of Pathology University of British Columbia Vancouver Canada; ^27^ Nantz National Alzheimer Center Houston Methodist Houston USA; ^28^ Department of Psychiatry and Behavioral Sciences Johns Hopkins University School of Medicine Baltimore USA; ^29^ University of Colorado Denver Aurora USA; ^30^ Department of Neurology University of Alabama at Birmingham Sparks Center Birmingham USA; ^31^ Department of Neurology Healthy Aging & Alzheimer's Care Center University of Chicago Chicago USA; ^32^ Dementia Research Centre Department of Neurodegenerative Disease UCL Institute of Neurology Queen Square London UK; ^33^ Sunnybrook Health Sciences Centre Sunnybrook Research Institute Toronto Canada; ^34^ Alzheimer's Disease and Other Cognitive Disorders Unit Neurology Service Hospital Clínic, Institut d'Investigacións Biomèdiques August Pi I Sunyer University of Barcelona Barcelona Spain; ^35^ Clinique Interdisciplinaire de Mémoire Département des Sciences Neurologiques CHU de Québec, and Faculté de Médecine, Université Laval Quebec Canada; ^36^ Karolinska Institute Department NVS Centre for Alzheimer Research Division of Neurogenetics Stockholm Sweden; ^37^ Unit for Hereditary Dementias Theme Aging Karolinska University Hospital Stockholm Sweden; ^38^ Fondazione IRCCS Ospedale Policlinico Milano Italy; ^39^ University of Milan Centro Dino Ferrari Milano Italy; ^40^ Laboratory for Cognitive Neurology Department of Neurosciences KU Leuven Leuven Belgium; ^41^ Neurology Service University Hospitals Leuven Leuven Belgium; ^42^ Faculty of Medicine University of Lisbon Lisbon Portugal; ^43^ Fondazione IRCCS Istituto Neurologico Carlo Besta Via Giovanni Celoria Milano Italy; ^44^ University Hospital of Coimbra (HUC) Neurology Service Faculty of Medicine University of Coimbra Coimbra Portugal; ^45^ Centre of Neurosciences and Cell biology University of Coimbra Coimbra Portugal; ^46^ Division of Psychology Communication and Human Neuroscience Wolfson Molecular Imaging Centre University of Manchester Manchester UK; ^47^ Department of Nuclear Medicine Centre for Translational Neuro‐ and Behavioral Sciences University Medicine Essen Essen Germany; ^48^ Department of Geriatric Medicine Klinikum Hochsauerland Arnsberg Germany; ^49^ Department of Neurology Ludwig‐Maximilians Universität München Munich Germany; ^50^ Centre for Neurodegenerative Diseases (DZNE) Munich Germany; ^51^ Munich Cluster of Systems Neurology Munich Germany; ^52^ Department of Neurofarba University of Florence Firenze Italy; ^53^ IRCCS Fondazione Don Carlo Gnocchi Florence Italy; ^54^ Department of Neurology University of Ulm Ulm Germany; ^55^ University Lille Lille France; ^56^ Inserm 1172 Lille France; ^57^ CHU CNR‐MAJ Labex Distalz LiCEND Lille Lille France; ^58^ Douglas Mental Health University Institute Department of Psychiatry McGill University Montreal Canada; ^59^ McConnell Brain Imaging Centre Montreal Neurological Institute McGill University Montreal Canada; ^60^ Nuffield Department of Clinical Neurosciences Medical Sciences Division University of Oxford Headley Way, Headington Oxford UK; ^61^ Department of Brain Sciences Imperial College London UK, Burlington Danes The Hammersmith Hospital London UK; ^62^ Sorbonne Université Paris Brain Institute – Institut du Cerveau – ICM, Inserm U1127, CNRS UMR 7225, AP‐HP ‐ Hôpital Pitié‐Salpêtrière Paris France; ^63^ Reference Center for Rare or Early‐onset Dementias, IM2A Department of Neurology AP‐HP ‐ Pitié‐Salpêtrière Hospital Paris France; ^64^ Department of Neurology AP‐HP ‐ Pitié‐Salpêtrière Hospital Paris France; ^65^ Department of Clinical Neurological Sciences University of Western Ontario London Canada; ^66^ Department of Clinical Neurosciences and Cambridge University Hospitals NHS Trust, University of Cambridge Department of Clinical Neurosciences Cambridge Biomedical Campus Cambridge UK; ^67^ Department of Neurodegenerative Diseases Hertie‐Institute for Clinical Brain Research & Centre of Neurology University of Tübingen Tübingen Germany; ^68^ Centre for Neurodegenerative Diseases (DZNE) Tübingen Germany; ^69^ Cognitive Disorders Unit Department of Neurology Hospital Universitario Donostia San Sebastian Gipuzkoa Spain; ^70^ Neuroscience Area Biodonostia Health Research Institute San Sebastian Gipuzkoa Spain; ^71^ Neurology Unit Department of Clinical and Experimental Sciences University of Brescia Piazza del Mercato Brescia Italy

**Keywords:** behavioral variant frontotemporal dementia, clinical diagnosis, diversity, sex difference

## Abstract

**INTRODUCTION:**

Higher male prevalence in sporadic behavioral variant frontotemporal dementia (bvFTD) has been reported. We hypothesized differences in phenotypes between genetic and sporadic bvFTD females resulting in underdiagnosis of sporadic bvFTD females.

**METHODS:**

We included genetic and sporadic bvFTD patients from two multicenter cohorts. We compared behavioral and cognitive symptoms, and gray matter volumes, between genetic and sporadic cases in each sex.

**RESULTS:**

Females with sporadic bvFTD showed worse compulsive behavior (*p* = 0.026) and language impairments (*p* = 0.024) compared to females with genetic bvFTD (*n* = 152). Genetic bvFTD females had smaller gray matter volumes than sporadic bvFTD females, particularly in the parietal lobe.

**DISCUSSION:**

Females with sporadic bvFTD exhibit a distinct clinical phenotype compared to females with genetic bvFTD. This difference may explain the discrepancy in prevalence between genetic and sporadic cases, as some females without genetic mutations may be misdiagnosed due to atypical bvFTD symptom presentation.

**Highlights:**

- Sex ratio is equal in genetic behavioral variant of frontotemporal dementia (bvFTD), whereas more males are present in sporadic bvFTD.
- Distinct neuropsychiatric phenotypes exist between sporadic and genetic bvFTD in females.
- Phenotype might explain the sex ratio difference between sporadic and genetic cases.

## 1 INTRODUCTION

Frontotemporal dementia (FTD) encompasses a spectrum of clinical syndromes that are associated with frontotemporal lobar degeneration (FTLD). These varying syndromes include behavioral variant of FTD (bvFTD),^1^ nonfluent and semantic variant primary progressive aphasia (PPA),^2^, ^3^ progressive supranuclear palsy (PSP),^4^ corticobasal syndrome,^5^, ^6^ and FTD with motor neuron disease also known as amyotrophic lateral sclerosis (FTD‐ALS).^7^ The heritability of clinical FTD syndromes varies, with notable differences across cohorts and geographical regions. FTD‐ALS and bvFTD consistently show the highest heritability, ranging from 10% to 40%. In contrast, PPA consistently exhibits the lowest heritability at less than 5%.^8^ The majority of FTD cases, however, lack monogenetic causes and are often referred to as sporadic FTD.^9^ bvFTD is the most common clinical subtype, and both genetic bvFTD and sporadic bvFTD are characterized by behavioral features of disinhibition, apathy, loss of empathy, compulsive behavior, hyperorality, and executive function decline.^1^ Although bvFTD affects both sexes, emerging research suggests the existence of sex‐linked differences in its clinical presentation, disease course, and cognitive reserve.^10^, ^11^, ^12^

The incidence of bvFTD demonstrates a notable sex difference, with a higher incidence of males than females.^13^ Because the sex distribution is balanced in genetic bvFTD, it is hypothesized that the reported sex imbalance in bvFTD may be attributed to a male predominance in sporadic bvFTD.^14^ Although the reasons behind this observed sex difference are just beginning to be explored, they likely stem from a combination of biological and sociocultural factors.^15^ From a biological perspective, for example, females with bvFTD show greater brain atrophy burden than males while showing similar cognitive and functional impairment at a similar age of diagnosis, implying the existence of greater behavioral reserve in females.^11^ From a sociocultural perspective, the higher frequency of initial psychiatric misdiagnosis reported in females presenting with behavioral change who were ultimately diagnosed with bvFTD^16^ could stem from a referral bias of women with behavioral disorders. This bias could ultimately lead to the underrepresentation of females in sporadic bvFTD cohorts. In contrast, a female presenting with behavioral alterations alongside a positive family history of bvFTD is likely to be referred to a specialized memory or FTD clinic early after symptom onset, leading to a more equal representation of both sexes in genetic bvFTD cohorts.

Over time, research has shown that bvFTD symptoms extend beyond the clinical hallmarks present in the consensus criteria,^1^ indicating involvement of neuropsychiatric features such as delusions, hallucinations, and depression.^17^ Notably, the nature, prevalence, and severity of these neuropsychiatric symptoms in bvFTD appear to diverge between sexes.^10^, ^18^ Because the equal sex distribution in genetic bvFTD may be due to a shielding effect of a positive family history against misclassification of neuropsychiatric features in bvFTD as psychiatric disorders, we hypothesize that a neuropsychiatric clinical subtype may currently be overlooked or misclassified as a psychiatric disorder in females with sporadic bvFTD. Therefore, the aim of this study is to investigate the discrepancy in the prevalence of bvFTD between genetic and sporadic cases in females by identifying distinct neuropsychiatric subtypes in genetic bvFTD and testing which subtype(s) is not or poorly represented in females with sporadic bvFTD.

## 2 METHODS

### 2.1 Participants

This study included patients with a diagnosis of probable or possible bvFTD from two international multicenter cohorts, the Advancing Research and Treatment in Frontotemporal Lobar Degeneration (ARTFL) and Longitudinal Evaluation of Familial Frontotemporal Dementia Subjects (LEFFTDS) Longitudinal Frontotemporal Lobar Degeneration (ALLFTD, previously known as ARTFL and LEFFTDS consortia) study and the Genetic Frontotemporal dementia Initiative (GENFI) study.

The ALLFTD study enrolled participants through a consortium of 27 centers across the United States and Canada between 2015 and 2023. Here we report data from the baseline measure for each participant as of December 2023. This study involves human participants and obtained ethical approval at each site. All participants provided written informed consent or assent with proxy consent. The ALLFTD consists of sporadic bvFTD patients and bvFTD patients carrying pathogenic genetic mutations in chromosome 9 open reading frame 72 (*C9orf72)*, microtubule‐associated protein tau (*MAPT)*, progranulin (*GRN)*, and other known FTD genetic mutations. Clinical diagnoses were made by clinicians experienced in FTD, based on medical history review, mental status examination, and a neurological examination. Examinations included disease severity measured by the Clinical Dementia Rating (CDR) Dementia Staging Instrument plus the Sum of Boxes score of Behavior and Language domains from the National Alzheimer's Coordinating Center FTLD Module (CDR plus NACC FTLD‐SB),^19^ and comprehensive behavioral and cognitive assessments. Participants with a structural brain lesion or other neurologic disorder that could impact findings (e.g., multiple sclerosis) were excluded. More details of participant inclusion and exclusion criteria can be found in previous publications.^20^, ^21^

The GENFI is an international multicenter cohort study across Europe and Canada. GENFI recruited participants with genetic mutations of FTD and their relatives.^22^, ^23^ Here we report data from GENFI Data Freeze 6. Participants included carriers of genetic mutations in *C9orf72, GRN*, and *MAPT*, who have or have not shown symptoms, and their relatives without genetic mutations. Most participants are unaware of their genetic status at recruitment and remain unaware of their genetic status by a genetic‐guardianship process. Participants underwent a standardized clinical assessment consisting of a medical history, family history, and physical examination. Symptomatic status for bvFTD was based on the assessment by clinicians to determine whether the participants fulfilled the diagnostic criteria.^1^, ^2^, ^24^ Participants in the GENFI study who are not carriers of any known FTD genetic mutations were included as healthy controls in neuroimaging analysis.

**RESEARCH IN CONTEXT**

1. **Systematic review**: The authors reviewed systematically the literature using PubMed, preprint repositories, and research citing key articles. Emerging research suggests the existence of sex‐linked differences in the clinical presentation, disease course, and cognitive reserve of behavioral variant frontotemporal dementia (bvFTD). Although sex distribution is equal in genetic bvFTD, a lower female prevalence has been reported in sporadic bvFTD.
2. **Interpretation**: Our results indicate that females with sporadic bvFTD exhibit a distinct clinical phenotype compared to both females with genetic bvFTD and males with sporadic bvFTD. This difference may help to explain the lower prevalence of bvFTD among females in sporadic cases, as some females with sporadic bvFTD may be misdiagnosed due to a lack of typical bvFTD symptoms, which align more closely with sporadic male bvFTD profiles.
3. **Future directions**: Future research should explore sex‐specific symptom evaluation and clinical diagnosis for bvFTD.

Demographics of all participants (*N* = 738) are shown in Table 1. In order to compare multiple variables, participants with missing data in any variable of interest were excluded. Participants with available behavioral and cognitive data were included in the analysis of behavioral and cognitive symptoms (*n* = 450, demographics presented in Table S1). Participants with one or more missing behavioral or cognitive variables were excluded from the analysis (*n* = 288). Comparison of the participants that were excluded and the participants that were included in analysis is shown in Figure S1. These two groups of participants did not differ significantly in their age [Kruskal‐Wallis test statistic (*H)*= 2.67, *p* = .10, effect size (*r*) = .060], age at disease onset (*H* = 2.59, *p* = .11, *r* = .059), or education (*H* = 1.36, *p* = .24, *r* = .043). However, participants who were excluded from the analysis had greater disease severity (*H* = 112.66, *p* < .001, *r *= .39) as assessed by the CDR plus NACC FTLD‐SB. Participants with available good quality structural magnetic resonance imaging (MRI) scans were included in voxel‐based morphometry analysis (*n* = 378, demographics presented in Table S2). Participants with missing scans or poor‐quality images before or after processing were excluded from voxel‐based morphometry analysis (*n* = 360). Comparison of the participants who were excluded and the participants who were included in analysis is shown in Figure S2. These two groups of participants were not significantly different in their age at disease onset (*H* = 2.35, *p* = .13, *r* = .056) or education (*H* = 2.01, *p* = .16, *r* = .052). Participants who were excluded from voxel‐based morphometry analysis had greater disease severity (*H* = 9.06, *p* = .0026, *r* = .11) as assessed by the CDR plus NACC FTLD‐SB. Participants who were excluded were also slightly older in age (*H* = 4.34, *p* = .040, *r* = .077). For analysis of other Neuropsychiatric Inventory Questionnaire (NPI‐Q) scores, participants with missing data in any of the NPI‐Q variables were excluded (*n* = 81). All other participants (*n* = 657) were included in the analysis of these NPI‐Q scores. The reason for using a different number of participants in the clinical and imaging analyses was to maximize the sample size in each analysis.

**TABLE 1:** Characteristics of participants stratified by sex.

	Total	Female	Male	Difference between females and males (chi‐square or Kruskal‐Wallis test)
**Participants N**	738	295	443	
**Diagnosis**	Probable bvFTD	252 (85.4%)	359 (81.0%)	*χ* ^2 ^= 2.09, *p* = .15, OR = 0.73 (95% CI: 0.49–1.09)
	Possible bvFTD	43 (14.6%)	84 (19.0%)	
**Type**	Sporadic	154 (52.2%)	273 (61.6%)	*χ* ^2 ^= 6.07, ***p* = .014**, OR = 1.47 (95% CI: 1.09–1.98)
	Genetic	141 (47.8%)	170 (38.4%)	
**Genetic mutation** **n (%)**	*C9orf72*	67 (47.5%)	88 (51.7%)	*H* = 0.57, *p* = .45, *r* = .028
	*GRN*	32 (22.7%)	37^a^ (21.8%)	
	*MAPT*	39 (27.7%)	37 (21.8%)	
	Other	3 (2.1%)	8 (4.7%)	
**Age (mean ± SD)**		62.3 ± 9.4	62.8 ± 8.5	*H* = 0.27, *p* = .61, *r* = .019
**Age at disease onset (mean ± SD)**		57.0 ± 9.8	57.2 ± 9.0	*H* = 0.00035, *p* = .99, *r* = .00069
**Education (years)**		15.0 ± 3.0	15.2 ± 3.1	*H* = 0.44, *p* = .50, *r* = .025
**CDR plus NACC FTLD‐SB (mean ± SD)**		10.2 ± 5.9	8.5 ± 4.4	*H* = 10.60, ***p* = .0011**, *r* = .12

*p* values in bold are statistically significant.

Abbreviations: CDR plus NACC FTLD‐SB, Clinical Dementia Rating Dementia Staging Instrument plus the Sum of Boxes score of Behavior and Language domains from the National Alzheimer's Coordinating Center FTLD Module; CI, confidence interval; C9orf72, chromosome 9 open reading frame 72; GRN, progranulin; MAPT, microtubule‐associated protein tau; OR, odds ratio; SD, standard deviation.

^a^ One subject has both *C9orf72* and *GRN* mutations.

### 2.2 Behavioral and cognitive assessment

The NPI‐Q^25^ was used to assess the presence and severity of neuropsychiatric and behavioral features. The NPI‐Q was completed with the informant and includes the following items: apathy, depression, delusions, hallucinations, disinhibition, irritability, agitation, anxiety, nighttime behavior, euphoria, motor disturbance, and appetite/eating behavior changes. All features were marked as present (mild, moderate, or severe) or absent. We primarily focused on the symptoms included in the FTD diagnostic criteria: apathy, disinhibition, eating behavior, loss of empathy, and compulsive behavior in addition to a dysexecutive neuropsychological profile.^1^ We also examined other NPI‐Q features available in both cohorts, including agitation, irritability, aberrant motor behavior, nighttime behavior, euphoria, delusions, hallucinations, depression, and anxiety. We created standard scores (z‐scores) for each variable within each cohort and used the standardized scores in statistical analysis.

For cognitive symptoms, four main cognitive domains were examined: executive functions, memory, language, and visuospatial memory. Due to the differences in cognitive tests between the two cohorts, standardized scores were first created for each variable within each cohort and a composite score was created for each cognitive domain with equal weight of each cognitive test. The sum of the composite scores from two cohorts was used. The cognitive tests used for the assessment of each cognitive domain are shown in Table 2.

**TABLE 2:** Cognitive assessments across domains in the ALLFTD and the GENFI cohorts.

	Executive functions	Memory	Language	Visuospatial
**ALLFTD**	Digit span forward^25^	Benson Complex Figure recall^26^	Category fluency (animals)^27^	Benson Complex Figure copy^28^
	Digit span backward^25^	Craft Story 21 Recall (immediate)^29^	Phonemic fluency (F)^27^	
	Trail Making Test Part A^30^	Craft Story 21 Recall (delayed)^29^	Multilingual Naming Test (total score)^31^	
	Trail Making Test Part B^30^			
**GENFI**	Digit span forward	Benson Complex Figure recall	Category fluency (animals)	Benson Complex Figure copy
	Digit span backward	Free and Cued Selective Reminding Test (FCSRT) free and total recall^26^	Phonemic fluency (F)	
	Trail Making Test Part A	FCSRT delayed free and total recall^26^	Boston Naming Test (short 30‐item version)^25^	
	Trail Making Test Part B			

### 2.3 Neuroimaging acquisition and processing

For MRI from the ALLFTD cohort, T1‐weighted MRI scans were collected on 3T scanners from one of three vendors: Siemens, Philips Medical System, or General Electric Medical Systems. A standard imaging acquisition protocol was used at all centers, managed, and reviewed for quality by a core group at the Mayo Clinic, Rochester. A T1‐weighted three‐dimensional (3D) magnetization prepared rapid gradient echo (MPRAGE) sequence was used to obtain the T1‐weighted images, with parameters as follows: 240 × 256 × 256 matrix; about 170 slices; voxel size = 1.05 × 1.05 × 1.25 mm^3^; flip angle, repetition time, and echo time varied by vendor.

For MRI from the GENFI cohort, T1‐weighted MRI scans were collected on 3T scanners from one of three vendors: Siemens, Philips Medical System, or General Electric Medical Systems. A T1‐weighted 3D MPRAGE sequence image was acquired for each subject accommodating different scanners at each site over at least 283 s (283–462 s) and had a median isotropic resolution of 1.1 mm (1–1.3 mm), repetition time of 2000 ms (6.6–2400), echo time of 2.9 ms (2.6–3.5 ms), inversion time of 8 ms (8–9 ms), and field of view (FOV) 256 × 256 × 208 mm (192 to 256 × 192 to 256 × 192 to 208 mm).

All T1‐weighted images were visually inspected for quality control. The acquired T1‐weighted images from both cohorts were processed using identical protocols in the Computational Anatomy Toolbox 12 (CAT12) (https://neuro‐jena.github.io/cat/) in Statistical Parametric Mapping (SPM) 12 (https://www.fil.ion.ucl.ac.uk/spm/software/spm12/) based on MATLAB (version 2023b). Images were denoised and corrected for intensity non‐uniformities and segmented into different tissue classes. Images were then spatially normalized using Diffeomorphic Anatomical Registration Through Exponentiated Lie Algebra (DARTEL) algorithm and registered to the Montreal Neurological Institute (MNI) 152 template. The resultant image in MNI space was modulated using the Jacobian determinants.^26^ Finally, images were spatially smoothed with an 8 mm full‐width at half maximum (FWHM) Gaussian kernel. The volumes of gray matter, white matter, and cerebrospinal fluid of the whole brain and the total intracranial volume (TIV) were calculated based on the segmented maps, as part of the CAT 12 preprocessing. All processed gray matter images were inspected one‐by‐one visually for image quality assurance before further analysis. In addition, the weighted image quality rating (IQR) provided by CAT12 were compared across different scanner types and cohorts to ensure consistency in data quality. The average post‐processing IQR across scanner types and cohorts of the participants included in voxel‐based morphometry analysis are shown in Figure S3.

### 2.4 Voxel‐based morphometry

Voxel‐based morphometry analysis was performed in SPM to compare the global gray matter volume (GMV) (1) between sporadic and genetic bvFTD stratified by sex (male/female); and (2) between females and males stratified by genetic status (sporadic/genetic). Age and TIV were included for correction in the analysis. Disease severity was included as a covariate for comparison between patient groups. MRI scanner type was included as an additional covariate to correct for any difference due to scanners in supplementary analysis. The smoothed and modulated images, GMV, were analyzed using voxel‐wise two‐sample *t*‐tests. Results were corrected for multiple comparisons by family‐wise error (FWE) correction. Clusters with FWE‐corrected *p* value < .05 were considered statistically significant.

### 2.5 Statistical analysis

Multiple linear regression was used to investigate the relationship between sex and the scores of behavioral and cognitive tests; and the relationship between type of bvFTD (i.e., sporadic vs genetic) and the scores of behavioral and cognitive tests. Each test score was used as the dependent variable in a regression model, and sex or type was used as the independent variable with education, age at disease onset, and disease severity measured by CDR plus NACC FTLD‐SB as covariates. The Benjamini and Hochberg procedure was used for controlling the false discovery rate (FDR).^27^ An FDR‐corrected *p* value < .05 was considered statistically significant. Demographics were compared between groups using the chi‐square test for categorical variables and the Kruskal–Wallis test for continuous variables, as variables were not normally distributed. The odd ratios (ORs), 95% confidence interval (CI), and effect size (*r*) were reported in results.

## 3 RESULTS

### 3.1 Participant characteristics across groups

Characteristics of participants stratified by sex are shown in Table 1. There was no significant difference between females and males in the ratio of probable/possible bvFTD diagnosis (*χ* ^2 ^= 2.09, *p* = .15, OR = 0.73 [95% CI: 0.49–1.09]), the ratio of each genetic mutation type (*H* = 0.57, *p* = .45, *r* = .028), age (*H* = 0.27, *p* = .61, *r* = .019), age at disease onset (*H* = 0.00035, *p* = .99, *r* = .00069), or education (*H* = 0.44, *p* = .50, *r* = .025). Females presented greater disease severity as measured by CDR plus NACC FTLD‐SB compared to males (*H* = 10.60, *p* = .0011, *r* = .12). To compare between sporadic and genetic groups, Table 3 shows the characteristics stratified by bvFTD type (i.e., sporadic vs genetic). There was a significant difference in sex ratio between sporadic and genetic bvFTD (*χ* ^2 ^= 6.07, *p* = .014, OR = 1.47 [95% CI: 1.09–1.98]). Forty‐five percent of the genetic bvFTD was female, whereas in sporadic bvFTD 35% was female. The difference in sex ratio was more apparent within the ALLFTD cohort (*χ* ^2 ^= 12.07, *p* < .001, OR = 1.93 [95% CI: 1.34–2.78]), in which there were 52% of genetic bvFTD was female, whereas 36% of sporadic bvFTD was female. Sporadic bvFTD patients were older (*H* = 16.64, *p* < .001, *r* = .15) and had a higher age at disease onset (*H* = 22.36, *p* < .001, *r* = .17) compared to genetic bvFTD patients. There was no significant difference in disease severity as measured by CDR plus NACC FTLD‐SB between sporadic and genetic bvFTD (*H* = 1.58, *p* = .21, *r* = .046).

**TABLE 3:** Characteristics of participants stratified by bvFTD type.

	Sporadic	Genetic			Difference between sporadic and genetic cases (chi‐square or Kruskal‐Wallis test)	
Cohort	ALLFTD	Total genetic	ALLFTD	GENFI	Sporadic vs genetic	Sporadic vs genetic (ALLFTD)
**Participants N**						
738	427	311	165	146		
**Sex** **n (%)**						
Female	154 (36.1%)	141 (45.3%)	86 (52.1%)	55 (37.7%)	*χ* ^2 ^= 6.07, ***p* = .014**, OR = 1.47 (95% CI: 1.09–1.98)	*χ* ^2 ^= 12.07, ***p* <** **.001**, OR = 1.93 (95% CI: 1.34–2.78)
Male	273 (63.9%)	170 (54.7%)	79 (47.9%)	91 (62.3%)		
**Genetic mutation** **n (%)**						
*C9orf72*	/	155 (49.8%)	77 (46.7%)	78 (53.4%)	/	/
*GRN*	/	69^a^ (22.3%)	32^a^ (19.4%)	37 (25.3%)		
*MAPT*	/	76 (24.4%)	47 (28.5%)	29 (19.9%)		
Other	/	11 (3.5%)	9 (5.4%)	2 (1.4%)		
**Age (mean ± SD)**	63.8 ± 8.6	61.0 ± 9.1	60.3 ± 9.1	61.8 ± 9.0	*H* = 16.64, ***p* <** **.001**, *r* = .15	*H* = 16.97, ***p* <** **.001**, *r* = .15
**Age of disease onset (mean ± SD)**	58.5 ± 8.6	55.1 ± 10.0	53.8 ± 10.4	56.8 ± 9.2	*H* = 22.36, ***p* <** **.001**, *r* = .17	*H* = 26.07, ***p* <** **.001**, *r* = .19
**Education (years)**	15.9 ± 2.6	14.0 ± 3.4	15.4 ± 2.6	12.6 ± 3.6	*H* = 51.00, ***p* <** **.001**, *r* = .26	*H* = 3.55, *p* < .060, *r* = .069
**CDR plus NACC FTLD‐SB (mean ± SD)**	8.9 ± 4.5	9.7 ± 5.8	9.2 ± 5.5	10.3 ± 6.1	*H* = 1.58, *p* = .21, *r* = .046	*H* = 0.043, *p* = .84, *r* = .0077

*p* values in bold are statistically significant.

Abbreviations: CDR plus NACC FTLD‐SB, Clinical Dementia Rating Dementia Staging Instrument plus the Sum of Boxes score of Behavior and Language domains from the National Alzheimer's Coordinating Center FTLD Module; CI, confidence interval; C9orf72, chromosome 9 open reading frame 72; GRN, progranulin; MAPT, microtubule‐associated protein tau; OR, odds ratio; SD, standard deviation.

^a^ One subject has both *C9orf72* and *GRN* mutations.

### 3.2 Sex differences in behavioral and cognitive symptoms between sporadic and genetic bvFTD

**FIGURE 1:**
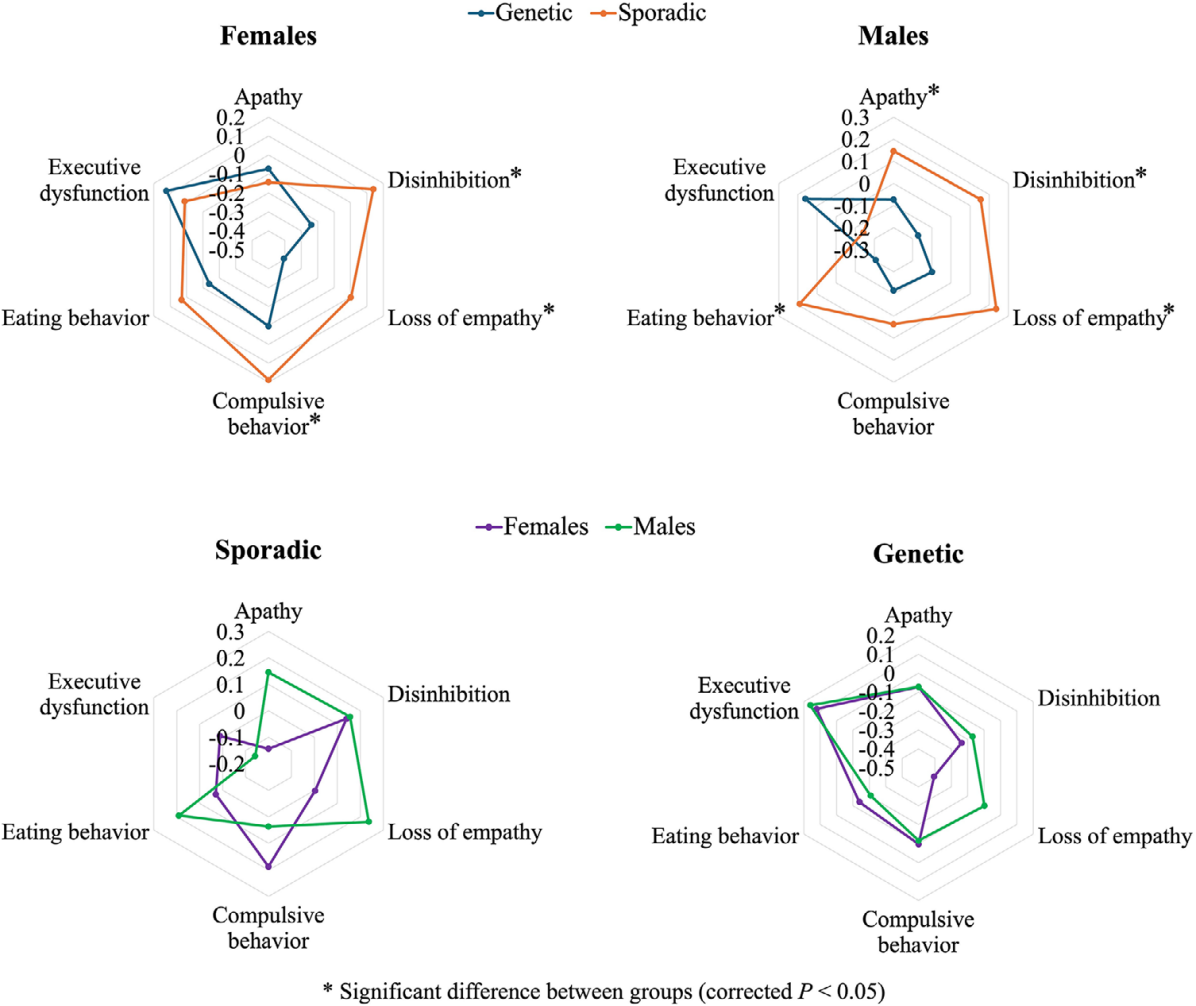
Differences in symptoms of frontotemporal dementia diagnostic criteria between genetic and sporadic bvFTD, stratified by sex; and differences in symptoms of frontotemporal dementia diagnostic criteria between females and males, stratified by bvFTD type. Values are *z*‐scores and higher values represent more severe symptoms. bvFTD, behavioral variant frontotemporal dementia.

Overall, sporadic bvFTD exhibited worse symptoms than genetic bvFTD, in both males and females (Figure 1). Females with sporadic bvFTD exhibited significantly more severe symptoms of compulsive behavior [*F* (4, 147) = 7.73, β = .39, *p* = .026], loss of empathy [*F* (4, 147) = 7.29, β = .45, *p* = .022], and disinhibition [*F* (4, 147) = 8.34, β = .38, *p* = .036] compared to females with genetic bvFTD. Males with sporadic bvFTD exhibited significantly more severe symptoms of apathy [*F* (4, 293) = 8.53, β = .35, *p *= .0050], eating behavior change [*F* (4, 293) = 10.75, β = .54, *p* < .001], loss of empathy [*F* (4, 293) = 10.11, β = .40, *p* = .0034], and disinhibition [*F* (4, 293) = 6.54, β = .39, *p* = .0034] compared to males with genetic bvFTD (Figure 1). No significant difference was found between females and males within the sporadic cases or within the genetic cases.

**FIGURE 2:**
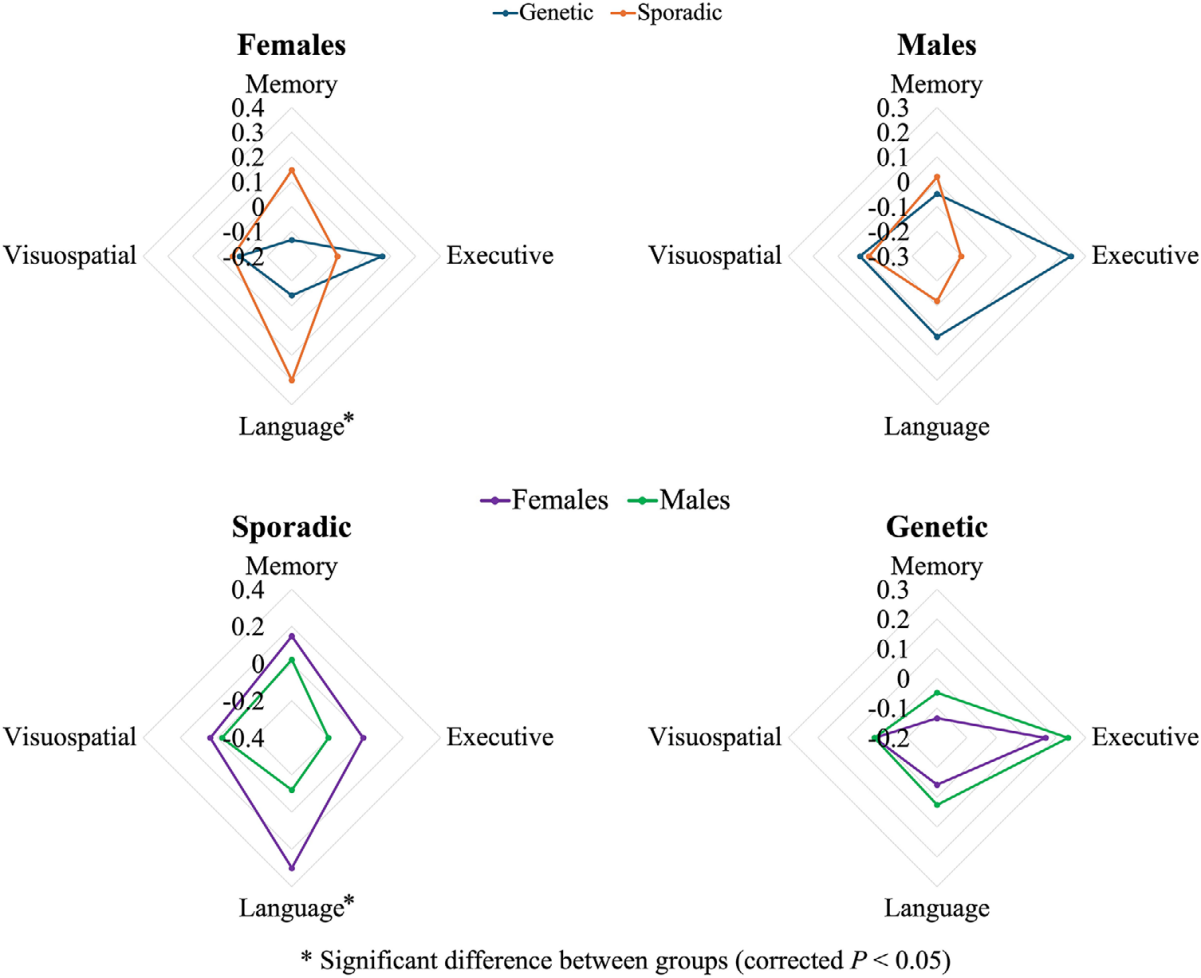
Differences in cognitive symptoms between genetic and sporadic bvFTD, stratified by sex; and differences in cognitive symptoms between females and males, stratified by bvFTD type. Each cognitive domain was assessed by the composite score of cognitive tests included for that domain as listed in Table 2. Values are *z*‐scores and higher values represent more severe symptoms. bvFTD, behavioral variant frontotemporal dementia.

In regard to cognitive functions, females with sporadic bvFTD showed more severe language impairments [*F* (4, 147) = 12.49, β = .43, *p* = .024] compared to females with genetic bvFTD (Figure 2). When compared between females and males within the sporadic cases, females showed more severe language impairments than males [*F* (4, 249) = 22.12, β = .42, *p* = .0042] (Figure 2). This was supported by a significant interaction between bvFTD type (sporadic/genetic) and sex in predicting language impairments (*p* = .029). No significant difference was found between females and males within the genetic cases.

**FIGURE 3:**
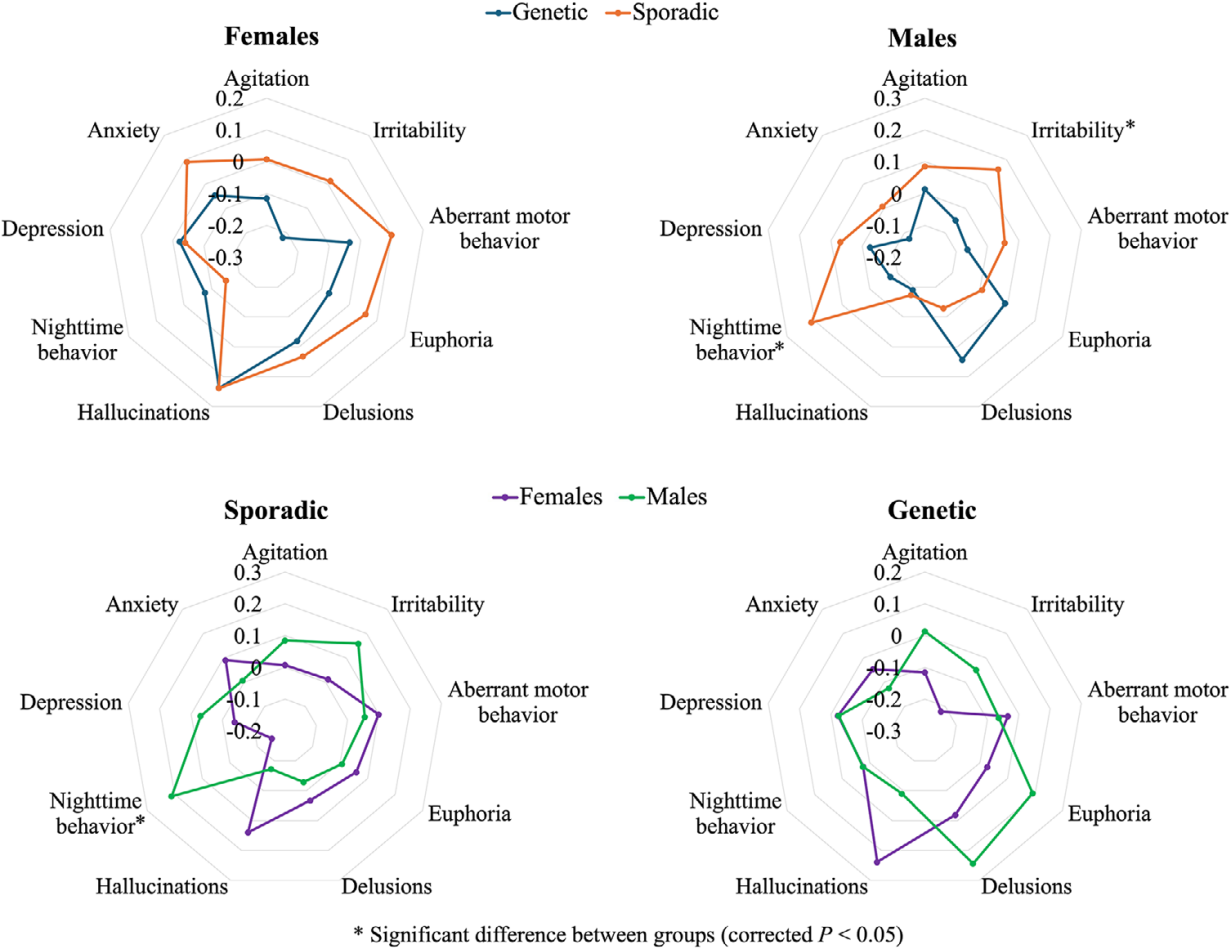
Differences in symptoms estimated by the NPI‐Q scores in genetic bvFTD and sporadic bvFTD, stratified by sex; and differences in symptoms estimated by the NPI‐Q scores between females and males, stratified by bvFTD type. Values are *z*‐scores and higher values represent more severe symptoms. bvFTD, behavioral variant frontotemporal dementia; NPI‐Q, Neuropsychiatric Inventory Questionnaire.

Other symptoms estimated by NPI‐Q scores are shown in Figure 3. Males with sporadic bvFTD exhibited significantly more severe symptoms of nighttime behavior [*F* (4, 390) = 5.74, β = .38, *p* = .0052] and irritability [*F* (4, 390) = 5.07, β = .34, *p* = .0052] compared to males with genetic bvFTD. No significant difference was found in females between sporadic bvFTD and genetic bvFTD. When compared between females and males within the sporadic cases, males showed more severe symptoms of nighttime behavior compared to females [*F* (4, 373) = 5.69, β = .41, *p* = .0015] (Figure 3). This was supported by a significant interaction between bvFTD type (sporadic/genetic) and sex in predicting nighttime behavior (*p* = .017). No significant difference was found between females and males within the genetic cases.

### 3.3 Sex differences in GMV between sporadic and genetic bvFTD

**FIGURE 4:**
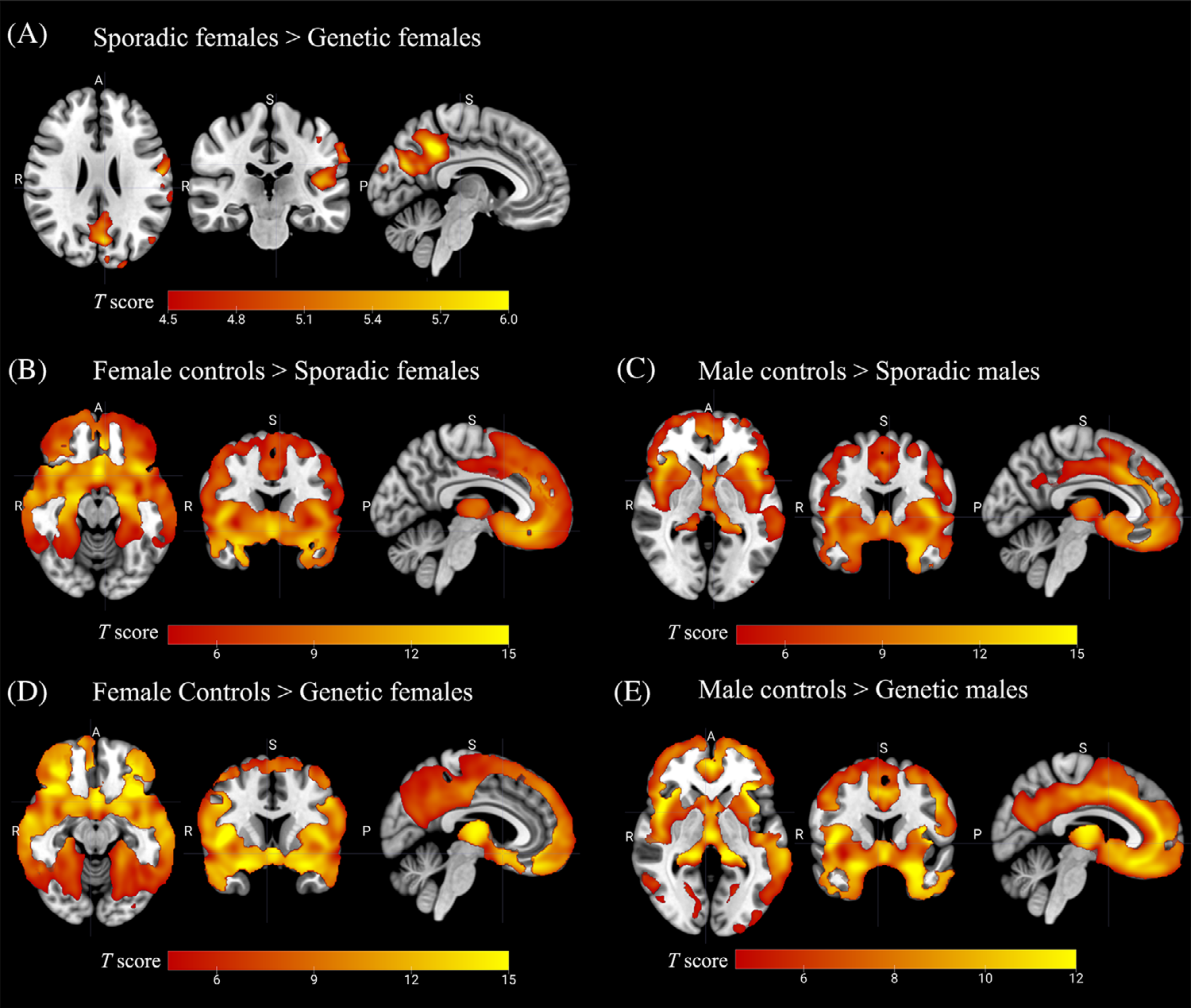
Voxel‐based morphometry analysis results of gray matter volume comparison between (A) females with sporadic bvFTD and females with genetic bvFTD (no significant difference was found in males between sporadic and genetic bvFTD); (B) female controls and females with sporadic bvFTD; (C) male controls and males with sporadic bvFTD; (D) female controls and females with genetic bvFTD; (E) male controls and males with genetic bvFTD. Results are showing the regions with significant differences (corrected *p* < .05). bvFTD, behavioral variant frontotemporal dementia.

Females with sporadic bvFTD showed greater GMV than females with genetic bvFTD, mainly in the left precuneus, left postcentral gyrus, left angular gyrus, left inferior occipital gyrus, and left inferior occipital gyrus (Figure 4). No significant difference was found in males between sporadic and genetic bvFTD or between females and males within the sporadic or genetic groups. Results did not change significantly when including scanner type as an additional covariate in the analysis (Figure S4). Results were also consistent when including cohort or disease duration as an additional covariate in the analysis.

## 4 DISCUSSION

This study investigated the discrepancy in the prevalence of females between genetic and sporadic bvFTD by identifying the impact of biological sex on clinical phenotypes. Patients with sporadic bvFTD generally had worse symptoms than patients with genetic bvFTD, despite similar disease stages, suggesting a potentially higher symptom threshold for diagnosis in sporadic bvFTD. Females and males were similar in genetic bvFTD, but females presented greater language impairments, whereas males presented worse nighttime behavior in sporadic bvFTD. In females only, sporadic cases showed more severe compulsive behavior and less parietal lobe atrophy than genetic cases. The discrepancy in prevalence of females between genetic and sporadic bvFTD might be explained by distinct neuropsychiatric subtypes, and some females without genetic mutations may be misdiagnosed due to fewer typical bvFTD symptoms from diagnostic criteria that may be more applicable to males with sporadic bvFTD.

There were different characteristics between sporadic and genetic bvFTD, regardless of sex. Sporadic participants were older and had a greater age at disease onset, consistent with existing literature showing that genetic FTLD tends to manifest at an earlier age than sporadic cases, and the age at onset is correlated with mean family age at onset.^28^ Sporadic and genetic bvFTD share core features, and no symptom has been reported exclusively in one type. Although sporadic and genetic patients in this study were at similar disease stages, sporadic patients showed worse symptoms of disinhibition and loss of empathy. This result suggests a potentially higher symptom threshold for diagnosis without known genetic mutations, although such discrepancy in symptoms does not necessarily result in a significant difference in disease severity rated by current clinical scale. The clinical syndromes and underlying pathology in sporadic bvFTD are more variable and less well understood compared to genetic bvFTD,^29^, ^30^ increasing challenges in accurate diagnosis and estimation of prevalence. Among the symptoms included in the diagnostic criteria, disinhibition and loss of empathy are generally more classic bvFTD symptoms along with apathy.^1^ In this study, prominent symptoms of disinhibition and loss of empathy in sporadic patients might aid in their identification and subsequent diagnosis.

Although the significance of sex in FTD has been increasingly reported,^10^, ^11^, ^12^, ^14^ the sex‐linked differences in clinical presentation and cognitive reserve remain poorly understood. We found that females showed less apathy and eating behavior disturbances, consistent with higher behavioral reserve of these symptoms in females reported in a previous study.^11^ However, language impairment was greater in females with sporadic bvFTD compared to females with genetic bvFTD and males with sporadic bvFTD. Although language problems are most prominent in PPA, it is generally considered a common symptom in bvFTD.^29^ As the sex ratio between sporadic and genetic cases in PPA is relatively equal,^14^ it is conceivable that the manifestation of language problems similar to the language variants of FTD is more rapidly picked up by family members and recognized by physicians. Thus, language impairments here might have facilitated differentiation from psychiatric disorders, preventing referral bias and misdiagnosis in these females.

Males with sporadic bvFTD exhibited more severe nighttime behavior than males with genetic bvFTD and females with sporadic bvFTD. There are several possible explanations for this finding. The NPI‐Q‐assessed nighttime behavior serves as a surrogate for sleep and nighttime disturbances.^31^ Sleep disturbances have been found in up to 85% of bvFTD and often manifest early in the disease course.^32^, ^33^, ^34^, ^35^ Nighttime behaviors of patients with bvFTD correlate strongly with caregiver distress.^34^ Clinical rating scores are susceptible to the sex of the caregiver.^36^ Female caregivers tend to report higher severity scores compared to male caregivers, even when patients are at similar biological stages of the disease.^36^ It is possible that female caregivers are more attuned to the subtle early signs, like nighttime behaviors, in male bvFTD patients. Second, sleep disturbances seen in rapid eye movement behavior disorder (RBD), which are associated with alpha‐synucleinopathies,^37^, ^38^ could be relevant, as alpha‐synucleinopathies and associated RBD symptoms are more prevalent in males.^39^ Although neuropathological data are lacking, it is possible that some males with sporadic bvFTD had more alpha‐synuclein co‐pathology, resulting in more sleep disturbances.^40^ Nevertheless, the current finding could also suggest that males with sporadic bvFTD present a different phenotype featuring more nighttime behaviors. Thus the more severe nighttime behaviors in males might be the result of a complex interplay between sex and co‐pathological mechanisms, which requires further research to fully understand.

Neurodegenerative disease is often misclassified as psychiatric disease due to overlapping symptoms, with bvFTD at the highest risk.^16^ A systematic review showed that ≈50% of patients with bvFTD received a prior psychiatric diagnosis and, more importantly, females received a prior psychiatric diagnosis more often than males in bvFTD.^16^ The most common initial psychiatric diagnosis that bvFTD patients received is major depressive disorder, possibly due to the similarity between some of their typical symptoms, such as apathy in bvFTD and social withdrawal in major depressive disorder.^41^, ^42^ As it is commonly known that the lifetime prevalence of major depressive disorder in females is almost two times that in males,^43^, ^44^ females with bvFTD are likely to be more susceptible to mistaken psychiatric diagnosis, especially those without genetic mutations known for FTD. In this study, the depression symptoms of females with sporadic bvFTD were relatively mild compared to other behavioral symptoms. This might be one of the major reasons that these females were not misdiagnosed as having major depressive disorder, whereas the portion of females with bvFTD who presented relatively severe symptoms of depression might have received psychiatric diagnosis. Sporadic males displayed more severe apathy than genetic males, whereas no difference was apparent between sporadic and genetic females, also suggesting that females with severe apathy might be misdiagnosed as having psychiatric disorder. Compulsive behavior, on the other hand, is more commonly present in bvFTD than in depression. Females with sporadic bvFTD exhibited more compulsive behavior than females with genetic bvFTD, which is another possible factor preventing these females from being misdiagnosed. As obsessive‐compulsive disorder usually arises earlier in life,^45^ compulsive behavior arising later in life is considered atypical for a psychiatric disorder. In addition, more pronounced apathy and eating behavior changes in males with sporadic bvFTD may support the notion that current diagnostic criteria are more closely aligned with sporadic male bvFTD profiles.

Atrophy patterns can be different between sporadic and genetic bvFTD and across genetic mutations.^46^, ^47^, ^48^ We found that genetic bvFTD females showed less GMV than sporadic bvFTD females, primarily in the left parietal lobe. This is consistent with literature showing greater atrophy in the parietal lobe of genetic bvFTD than sporadic bvFTD, mainly driven by the *C9orf72* mutation group.^46^ Of interest, the difference between genetic and sporadic cases was only significant in females—not in males. We speculate that the portion of females with sporadic bvFTD missed in diagnosis might present not only different neuropsychiatric symptoms, but also greater atrophy compared to those that had been identified in the current cohorts.

This study included participants from two cohorts with differing sex distributions in genetic bvFTD. The GENFI study primarily recruits participants from European countries, whereas the ALLFTD study recruits participants from North America. This discrepancy suggests a potential cultural effect on caregiver reporting. Although this topic is beyond the scope of the current study, future research could investigate whether cultural or regional factors influence the clinical reporting or diagnosis of genetic bvFTD.

This study has limitations. First, the exclusion of subjects with missing data reduced statistical power. This could be the primary reason that the interaction between sex and bvFTD type was insignificant in multiple regression predicting diagnostic criteria symptoms, even though different profiles were observed between genetic and sporadic cases across sexes. Participants who were excluded from analyses tended to have more advanced disease stages, likely because severe symptoms made it more challenging to collect complete data. This exclusion could introduce bias into the study by underrepresenting patients with more severe symptoms. Future studies could address this limitation by implementing robust imputation techniques and enhancing clinical protocols to facilitate data collection in this subgroup. Second, we integrated data from two multicenter cohorts. The variability of MRI acquisition scanners and sequences, cognitive tests, and clinical assessments is higher than a study using data from a single site. However, we mitigated the effects on neuroimaging through normalization, de‐noising, and standardized registration of MRI scans, and we also performed supplementary analyses to ensure that the results were not significantly affected by scanner type or cohort. We mitigated the effects on cognitive tests through standardizing test scores and creating composite scores.

In conclusion, there are sex‐linked differences in the clinical phenotypes of sporadic bvFTD. The discrepancy in prevalence of females between genetic and sporadic bvFTD might be attributable to misdiagnosis in females with sporadic bvFTD due to overlapping symptoms with psychiatric disorders. Because psychiatric misdiagnosis can lead to delayed and inappropriate treatment, clinicians should have more sex‐specific considerations when evaluating symptoms and making diagnosis.

## CONFLICT OF INTEREST STATEMENT

Dr. Litvan's research is supported by the National Institutes of Health (NIH) grants: 5U01NS112010/807745, U01NS100610, R25NS098999, U19 AG063911‐1 and 1R21NS114764‐01A1, and  2 P30 AG062429‐06; the Michael J Fox Foundation, Parkinson's Foundation, Roche, AbbVie, Lundbeck, EIP‐Pharma, Alterity, Novartis, and UCB. She is a member of the Scientific Advisory Board for the Rossy PSP Program at the University of Toronto, Aprinoia, Amydis, and the U.S. Food and Drug Administration (FDA) Peripheral and Central Nervous System Drugs Advisory Committee. She receives her salary from the University of California San Diego and as Chief Editor of *Frontiers in Neurology*. Eliana Marisa Ramos receives research support from the NIH. All other authors have no conflict of interest. Author disclosures are available in the Supporting Information.

## Supporting information



Supporting Information

Supporting Information

## 1

The GENFI Consortium Author List

**Table:**

Rhian Convery	Department of Neurodegenerative Disease, Dementia Research Centre, UCL Queen Square Institute of Neurology, London, UK
Martina Bocchetta	Department of Neurodegenerative Disease, Dementia Research Centre, UCL Queen Square Institute of Neurology, London, UK
David Cash	Department of Neurodegenerative Disease, Dementia Research Centre, UCL Queen Square Institute of Neurology, London, UK
Sophie Goldsmith	Department of Neurodegenerative Disease, Dementia Research Centre, UCL Queen Square Institute of Neurology, London, UK
Kiran Samra	Department of Neurodegenerative Disease, Dementia Research Centre, UCL Queen Square Institute of Neurology, London, UK
David L. Thomas	Neuroimaging Analysis Centre, Department of Brain Repair and Rehabilitation, UCL Institute of Neurology, Queen Square, London, UK
Thomas Cope	Cambridge University Hospitals NHS Trust, University of Cambridge, Cambridge, UK
Timothy Rittman	Department of Clinical Neurosciences, University of Cambridge, Cambridge, UK
Antonella Alberici	Centre for Neurodegenerative Disorders, Department of Clinical and Experimental Sciences, University of Brescia, Brescia, Italy
Enrico Premi	Stroke Unit, ASST Brescia Hospital, Brescia, Italy
Roberto Gasparotti	Neuroradiology Unit, University of Brescia, Brescia, Italy
Emanuele Buratti	ICGEB Trieste, Italy
Valentina Cantoni	Centre for Neurodegenerative Disorders, Department of Clinical and Experimental Sciences, University of Brescia, Brescia, Italy
Andrea Arighi	Fondazione IRCCS Ca’ Granda Ospedale Maggiore Policlinico, Neurodegenerative Diseases Unit, Milan, Italy
Chiara Fenoglio	University of Milan, Centro Dino Ferrari, Milan, Italy
Vittoria Borracci	Fondazione IRCCS Ca’ Granda Ospedale Maggiore Policlinico, Neurodegenerative Diseases Unit, Milan, Italy
Maria Serpente	Fondazione IRCCS Ca’ Granda Ospedale Maggiore Policlinico, Neurodegenerative Diseases Unit, Milan, Italy
Tiziana Carandini	Fondazione IRCCS Ca’ Granda Ospedale Maggiore Policlinico, Neurodegenerative Diseases Unit, Milan, Italy
Emanuela Rotondo	Fondazione IRCCS Ca’ Granda Ospedale Maggiore Policlinico, Neurodegenerative Diseases Unit, Milan, Italy
Giacomina Rossi	Fondazione IRCCS Istituto Neurologico Carlo Besta, Milano, Italy
Giorgio Giaccone	Fondazione IRCCS Istituto Neurologico Carlo Besta, Milano, Italy
Giuseppe Di Fede	Fondazione IRCCS Istituto Neurologico Carlo Besta, Milano, Italy
Paola Caroppo	Fondazione IRCCS Istituto Neurologico Carlo Besta, Milano, Italy
Sara Prioni	Fondazione IRCCS Istituto Neurologico Carlo Besta, Milano, Italy
Veronica Redaelli	Fondazione IRCCS Istituto Neurologico Carlo Besta, Milano, Italy
David Tang‐Wai	The University Health Network, Krembil Research Institute, Toronto, Canada
Ekaterina Rogaeva	Tanz Centre for Research in Neurodegenerative Diseases, University of Toronto, Toronto, Canada
Miguel Castelo‐Branco	Faculty of Medicine, ICNAS, CIBIT, University of Coimbra, Coimbra, Portugal.
Morris Freedman	Baycrest Health Sciences, Rotman Research Institute, University of Toronto, Toronto, Canada
Ron Keren	The University Health Network, Toronto Rehabilitation Institute, Toronto, Canada
Sandra Black	Sunnybrook Health Sciences Centre, Sunnybrook Research Institute, University of Toronto, Toronto, Canada
Sara Mitchell	Sunnybrook Health Sciences Centre, Sunnybrook Research Institute, University of Toronto, Toronto, Canada
Christen Shoesmith	Department of Clinical Neurological Sciences, University of Western Ontario, London, Ontario, Canada
Robart Bartha	Department of Medical Biophysics, The University of Western Ontario, London, Ontario, Canada; Centre for Functional and Metabolic Mapping, Robarts Research Institute, The University of Western Ontario, London, Ontario, Canada
Rosa Rademakers	Center for Molecular Neurology, University of Antwerp
Jackie Poos	Department of Neurology, Erasmus Medical Center, Rotterdam, Netherlands
Janne M. Papma	Department of Neurology, Erasmus Medical Center, Rotterdam, Netherlands
Lucia Giannini	Department of Neurology, Erasmus Medical Center, Rotterdam, Netherlands
Liset de Boer	Department of Neurology, Erasmus Medical Center, Rotterdam, Netherlands
Julie de Houwer	Department of Neurology, Erasmus Medical Center, Rotterdam, Netherlands
Rick van Minkelen	Department of Clinical Genetics, Erasmus Medical Center, Rotterdam, Netherlands
Yolande Pijnenburg	Amsterdam University Medical Centre, Amsterdam VUmc, Amsterdam, Netherlands
Benedetta Nacmias	Department of Neuroscience, Psychology, Drug Research and Child Health, University of Florence, Florence, Italy
Camilla Ferrari	Department of Neuroscience, Psychology, Drug Research and Child Health, University of Florence, Florence, Italy
Cristina Polito	Department of Biomedical, Experimental and Clinical Sciences “Mario Serio”, Nuclear Medicine Unit, University of Florence, Florence, Italy
Gemma Lombardi	Department of Neuroscience, Psychology, Drug Research and Child Health, University of Florence, Florence, Italy
Valentina Bessi	Department of Neuroscience, Psychology, Drug Research and Child Health, University of Florence, Florence, Italy
Mattias Nilsson	Department of Clinical Neuroscience, Karolinska Institutet, Stockholm, Sweden
Henrik Viklund	Karolinska University Hospital Huddinge
Melissa Taheri Rydell	Department of Neurobiology, Care Sciences and Society; Center for Alzheimer Research, Division of Neurogeriatrics,, Bioclinicum, Karolinska Institutet, Solna, Sweden; Unit for Hereditary Dementias, Theme inflammation and Aging, Karolinska University Hospital, Solna, Sweden
Vesna Jelic	Department of Neurobiology, Care Sciences and Society; Division of Clinical Geriatrics, Karolinska Institutet, Stockholm, Sweden; Cognitive clinic, Theme inflammation and Aging, Karolinska University Hospital, Solna, Sweden
Linn Öijerstedt	Department of Neurobiology, Care Sciences and Society; Center for Alzheimer Research, Division of Neurogeriatrics,, Bioclinicum, Karolinska Institutet, Solna, Sweden; Unit for Hereditary Dementias, Theme inflammation and Aging, Karolinska University Hospital, Solna, Sweden
Tobias Langheinrich	Division of Neuroscience and Experimental Psychology, Wolfson Molecular Imaging Centre, University of Manchester, Manchester, UK; Manchester Centre for Clinical Neurosciences, Department of Neurology, Salford Royal NHS Foundation Trust, Manchester, UK
Albert Lladó	Alzheimer's disease and Other Cognitive Disorders Unit, Neurology Service, Hospital Clínic, Barcelona, Spain
Anna Antonell	Alzheimer's disease and Other Cognitive Disorders Unit, Neurology Service, Hospital Clínic, Barcelona, Spain
Jaume Olives	Alzheimer's disease and Other Cognitive Disorders Unit, Neurology Service, Hospital Clínic, Barcelona, Spain
Mircea Balasa	Alzheimer's disease and Other Cognitive Disorders Unit, Neurology Service, Hospital Clínic, Barcelona, Spain
Nuria Bargalló	Imaging Diagnostic Center, Hospital Clínic, Barcelona, Spain
Sergi Borrego‐Ecija	Alzheimer's disease and Other Cognitive Disorders Unit, Neurology Service, Hospital Clínic, Barcelona, Spain
Ana Verdelho	Department of Neurosciences and Mental Health, Centro Hospitalar Lisboa Norte—Hospital de Santa Maria & Faculty of Medicine, University of Lisbon, Lisbon, Portugal
Carolina Maruta	Laboratory of Language Research, Centro de Estudos Egas Moniz, Faculty of Medicine, University of Lisbon, Lisbon, Portugal
Tiago Coelho	Faculty of Medicine, University of Lisbon, Lisbon, Portugal
Gabriel Miltenberger	Faculty of Medicine, University of Lisbon, Lisbon, Portugal
Frederico Simões do Couto	Faculdade de Medicina, Universidade Católica Portuguesa
Alazne Gabilondo	Cognitive Disorders Unit, Department of Neurology, Donostia University Hospital, San Sebastian, Gipuzkoa, Spain; Neuroscience Area, Biodonostia Health Research Insitute, San Sebastian, Gipuzkoa, Spain
Ioana Croitoru	Neuroscience Area, Biodonostia Health Research Insitute, San Sebastian, Gipuzkoa, Spain
Mikel Tainta	Neuroscience Area, Biodonostia Health Research Insitute, San Sebastian, Gipuzkoa, Spain
Myriam Barandiaran	Cognitive Disorders Unit, Department of Neurology, Donostia University Hospital, San Sebastian, Gipuzkoa, Spain; Neuroscience Area, Biodonostia Health Research Insitute, San Sebastian, Gipuzkoa, Spain
Patricia Alves	Neuroscience Area, Biodonostia Health Research Insitute, San Sebastian, Gipuzkoa, Spain; Department of Educational Psychology and Psychobiology, Faculty of Education, International University of La Rioja, Logroño, Spain
Benjamin Bender	Department of Diagnostic and Interventional Neuroradiology, University of Tübingen, Tübingen, Germany
David Mengel	Department of Neurodegenerative Diseases, Hertie‐Institute for Clinical Brain Research and Center of Neurology, University of Tübingen, Tübingen, Germany; Center for Neurodegenerative Diseases (DZNE), Tübingen, Germany
Lisa Graf	Department of Neurodegenerative Diseases, Hertie‐Institute for Clinical Brain Research and Center of Neurology, University of Tübingen, Tübingen, Germany
Annick Vogels	Department of Human Genetics, KU Leuven, Leuven, Belgium
Mathieu Vandenbulcke	Geriatric Psychiatry Service, University Hospitals Leuven, Belgium; Neuropsychiatry, Department of Neurosciences, KU Leuven, Leuven, Belgium
Philip Van Damme	Neurology Service, University Hospitals Leuven, Belgium; Laboratory for Neurobiology, VIB‐KU Leuven Centre for Brain Research, Leuven, Belgium
Rose Bruffaerts	Department of Biomedical Sciences, University of Antwerp, Antwerp, Belgium; Biomedical Research Institute, Hasselt University, 3500 Hasselt, Belgium
Koen Poesen	Laboratory for Molecular Neurobiomarker Research, KU Leuven, Leuven, Belgium
Pedro Rosa‐Neto	Translational Neuroimaging Laboratory, McGill Centre for Studies in Aging, McGill University, Montreal, Québec, Canada
Maxime Montembault	Douglas Research Centre, Department of Psychiatry, McGill University, Montreal, Québec, Canada
Agnès Camuzat	Sorbonne Université, Paris Brain Institute – Institut du Cerveau – ICM, Inserm U1127, CNRS UMR 7225, AP‐HP—Hôpital Pitié‐Salpêtrière, Paris, France
Alexis Brice	Sorbonne Université, Paris Brain Institute – Institut du Cerveau – ICM, Inserm U1127, CNRS UMR 7225, AP‐HP—Hôpital Pitié‐Salpêtrière, Paris, France; Reference Network for Rare Neurological Diseases (ERN‐RND)
Anne Bertrand	Sorbonne Université, Paris Brain Institute – Institut du Cerveau – ICM, Inserm U1127, CNRS UMR 7225, AP‐HP—Hôpital Pitié‐Salpêtrière, Paris, France; Inria, Aramis project‐team, F‐75013, Paris, France; Centre pour l'Acquisition et le Traitement des Images, Institut du Cerveau et la Moelle, Paris, France
Aurélie Funkiewiez	Sorbonne Université, Paris Brain Institute – Institut du Cerveau – ICM, Inserm U1127, CNRS UMR 7225, AP‐HP—Hôpital Pitié‐Salpêtrière, Paris, France; Centre de référence des démences rares ou précoces, IM2A, Département de Neurologie, AP‐HP—Hôpital Pitié‐Salpêtrière, Paris, France
Daisy Rinaldi	Sorbonne Université, Paris Brain Institute – Institut du Cerveau – ICM, Inserm U1127, CNRS UMR 7225, AP‐HP—Hôpital Pitié‐Salpêtrière, Paris, France; Centre de référence des démences rares ou précoces, IM2A, Département de Neurologie, AP‐HP—Hôpital Pitié‐Salpêtrière, Paris, France; Département de Neurologie, AP‐HP—Hôpital Pitié‐Salpêtrière, Paris, France
Dario Saracino	Sorbonne Université, Paris Brain Institute – Institut du Cerveau – ICM, Inserm U1127, CNRS UMR 7225, AP‐HP—Hôpital Pitié‐Salpêtrière, Paris, France; Centre de référence des démences rares ou précoces, IM2A, Département de Neurologie, AP‐HP—Hôpital Pitié‐Salpêtrière, Paris, France; Inria, Aramis project‐team, F‐75013, Paris, France
Olivier Colliot	Sorbonne Université, Paris Brain Institute – Institut du Cerveau – ICM, Inserm U1127, CNRS UMR 7225, AP‐HP—Hôpital Pitié‐Salpêtrière, Paris, France; Centre pour l'Acquisition et le Traitement des Images, Institut du Cerveau et la Moelle, Paris, France; Inria, Aramis project‐team, F‐75013, Paris, France
Sabrina Sayah	Sorbonne Université, Paris Brain Institute – Institut du Cerveau – ICM, Inserm U1127, CNRS UMR 7225, AP‐HP—Hôpital Pitié‐Salpêtrière, Paris, France
Catharina Prix	Neurologische Klinik, Ludwig‐Maximilians‐Universität München, Munich, Germany
Elisabeth Wlasich	Neurologische Klinik, Ludwig‐Maximilians‐Universität München, Munich, Germany
Olivia Wagemann	Neurologische Klinik, Ludwig‐Maximilians‐Universität München, Munich, Germany
Sonja Schönecker	Neurologische Klinik, Ludwig‐Maximilians‐Universität München, Munich, Germany
Alexander Maximilian Bernhardt	Neurologische Klinik, Ludwig‐Maximilians‐Universität München, Munich, Germany
Anna Stockbauer	Neurologische Klinik, Ludwig‐Maximilians‐Universität München, Munich, Germany
Jolina Lombardi	Department of Neurology, University of Ulm, Ulm
Sarah Anderl‐Straub	Department of Neurology, University of Ulm, Ulm, Germany
Adeline Rollin	CHU, CNR‐MAJ, Labex Distalz, LiCEND Lille, France
Gregory Kuchcinski	Univ Lille, France; Inserm 1172, Lille, France; CHU, CNR‐MAJ, Labex Distalz, LiCEND Lille, France
Maxime Bertoux	Inserm 1172, Lille, France; CHU, CNR‐MAJ, Labex Distalz, LiCEND Lille, France
Thibaud Lebouvier	Univ Lille, France; Inserm 1172, Lille, France; CHU, CNR‐MAJ, Labex Distalz, LiCEND Lille, France
Vincent Deramecourt	Univ Lille, France; Inserm 1172, Lille, France; CHU, CNR‐MAJ, Labex Distalz, LiCEND Lille, France
João Durães	Neurology Department, Centro Hospitalar e Universitario de Coimbra, Coimbra, Portugal
Marisa Lima	Neurology Department, Centro Hospitalar e Universitario de Coimbra, Coimbra, Portugal
Maria João Leitão	Centre of Neurosciences and Cell Biology, Universidade de Coimbra, Coimbra, Portugal
Maria Rosario Almeida	Faculty of Medicine, University of Coimbra, Coimbra, Portugal
Miguel Tábuas‐Pereira	Neurology Department, Centro Hospitalar e Universitario de Coimbra, Coimbra, Portugal; Faculty of Medicine, University of Coimbra, Coimbra, Portugal.
Sónia Afonso	Instituto Ciencias Nucleares Aplicadas a Saude, Universidade de Coimbra, Coimbra, Portugal
João Lemos	Faculty of Medicine, University of Coimbra, Coimbra, Portugal
