# Supplementary material for: Sex differences in clinical phenotypes of behavioral variant frontotemporal dementia
Source: Alzheimers Dement. 2025 Apr 25;21(4):e14608. doi: 10.1002/alz.14608 (PMC12022892; doi:10.1002/alz.14608)
Supplement: Supplementary file 1 — Supporting Information [file ALZ-21-e14608-s001.docx]

**Supplementary Table 1**. Characteristics of participants with available data for behavioral and cognitive tests.

|  |  | **Sporadic** | **Genetic** | | | **Difference between sporadic and genetic cases (Chi-squared or Kruskal-Wallis test)** | |
| --- | --- | --- | --- | --- | --- | --- | --- |
| **Cohort** |  | ALLFTD | Total Genetic | ALLFTD | GENFI | Sporadic vs. Genetic | Sporadic vs. Genetic (ALLFTD) |
| **Participants N** | 450 | 254 | 196 | 92 | 104 |  |  |
| **Sex**  **n (%)** | Female | 79 (31.1%) | 73 (37.2%) | 41 (44.6%) | 32 (30.8%) | *χ*^2^ = 1.60, *P* = 0.21, OR = 1.31 (95% CI: 0.89-1.95) | *χ*^2^ = 4.83, ***P* = 0.028**, OR = 1.78 (95% CI: 1.09-2.91) |
|  | Male | 175 (68.9%) | 123 (62.8%) | 51 (55.4%) | 72 (69.2%) |  |  |
| **Genetic mutation**  **n (%)** | *C9orf72* | / | 101 (51.5%) | 46 (50.0%) | 55 (52.9%) | / | / |
|  | *GRN* | / | 37 (18.9%) | 14 (15.2%) | 23 (22.1%) |  |  |
|  | *MAPT* | / | 53 (27.0%) | 29 (31.5%) | 24 (23.1%) |  |  |
|  | Other | / | 5 (2.6%) | 3 (3.3%) | 2 (1.9%) |  |  |
| **Age (mean±SD)** |  | 63.7±7.7 | 60.4±8.3 | 60.6±8.3 | 60.1±8.4 | *H* = 16.57, ***P* < 0.001**, *r* = 0.19 | *H* = 9.38, ***P* = 0.0022**, *r* = 0.16 |
| **Age of disease onset (mean±SD)** |  | 58.4±8.0 | 54.6±9.0 | 53.9±9.8 | 55.3±8.3 | *H* = 20.25, ***P* < 0.001**, *r* = 0.21 | *H* = 16.65, ***P* < 0.001**, *r* = 0.22 |
| **Education (years)** |  | 15.8±2.5 | 13.9±3.3 | 15.4±2.4 | 12.6±3.5 | *H* = 36.69, ***P* < 0.001**, *r* = 0.29 | *H* = 1.43, *P* = 0.23, *r* = 0.064 |
| **FTLDCDR-SB**  **(mean±SD)** |  | 7.1±3.2 | 8.1±4.6 | 6.8±3.3 | 9.3±5.3 | *H* = 2.49, *P* = 0.11, *r* = 0.074 | *H* = 0.81, *P* = 0.37, *r* = 0.048 |

Abbreviations: FTLDCDR-SB, Clinical Dementia Rating plus Frontotemporal Lobar Degeneration Behavior and Language Domains sum of boxes; CI, confidence interval; OR, odds ratio; SD, standard deviation


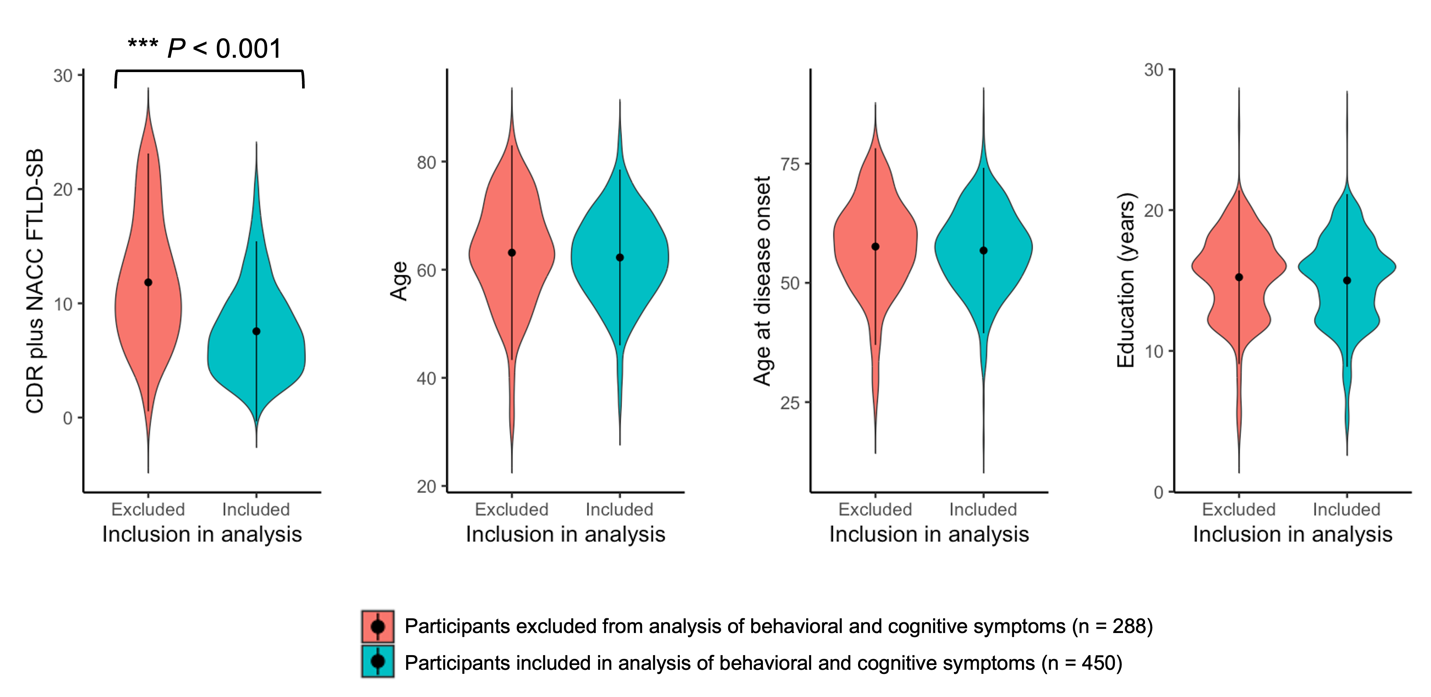


**Supplementary Figure 1**. Characteristics of participants with missing behavioral or cognitive data, which were therefore excluded from analysis (n = 288), and the rest of the participants which were included in the analysis of behavioral and cognitive symptoms (n = 450). These two groups of participants were not significantly different in their age (*H* = 2.67, *P* = 0.10, *r* = 0.060), age at disease onset (*H* = 2.59, *P* = 0.11, *r* = 0.059) or education years (*H* = 1.36, *P* = 0.24, *r* = 0.043). Participants that were excluded from analysis were more severe in disease (*H* = 112.66, *P* < 0.001, *r* = 0.39) as assessed by the Clinical Dementia Rating (CDR^®^) Dementia Staging Instrument plus the sum of boxes score of Behavior and Language domains from the National Alzheimer’s Coordinating Center FTLD Module (CDR plus NACC FTLD-SB).

**Supplementary Table 2**. Characteristics of participants with good quality T1-weighted structural MRI scans.

|  |  | **Sporadic** | **Genetic** | | | **Difference between sporadic and genetic cases (Chi-squared or Kruskal-Wallis test)** | |
| --- | --- | --- | --- | --- | --- | --- | --- |
| **Cohort** |  | ALLFTD | Total  Genetic | ALLFTD | GENFI | Sporadic vs. Genetic | Sporadic vs. Genetic (ALLFTD) |
| **Participants N** | 378 | 166 | 212 | 119 | 93 |  |  |
| **Sex**  **n (%)** | Female | 65 (39.2%) | 95 (44.8%) | 57 (47.9%) | 38 (40.9%) | *χ*^2^ = 1.00, *P* = 0.32, OR = 1.26 (95% CI: 0.83-1.91) | *χ*^2^ = 1.82, *P* = 0.17, OR = 1.43 (95% CI: 0.89-2.30) |
|  | Male | 101 (60.8%) | 117 (55.2%) | 62 (52.1%) | 55 (59.1%) |  |  |
| **Genetic mutation**  **n (%)** | *C9orf72* | / | 105 (49.5%) | 50 (42.0%) | 55 (59.1%) | / | / |
|  | *GRN* | / | 43 (20.3%) | 20 (16.8%) | 23 (24.7%) |  |  |
|  | *MAPT* | / | 57 (26.9%) | 43 (36.1%) | 14 (15.1%) |  |  |
|  | Other | / | 7 (3.3%) | 6 (5.1%) | 1 (1.1%) |  |  |
| **Age (mean±SD)** |  | 63.5±8.1 | 60.2±9.6 | 58.9±9.4 | 61.9±9.5 | *H* = 10.46, ***P* = 0.0012**, *r* = 0.17 | *H* = 15.63, ***P* < 0.001**, *r* = 0.23 |
| **Age of disease onset (mean±SD)** |  | 58.7±8.5 | 54.8±10.4 | 52.6±10.3 | 57.6±9.9 | *H* = 14.18, ***P* < 0.001**, *r* = 0.19 | *H* = 24.05, ***P* < 0.001**, *r* = 0.29 |
| **Education (years)** |  | 15.9±2.5 | 14.5±6.7 | 16.0±8.1 | 12.5±3.5 | *H* = 26.95, ***P* < 0.001**, *r* = 0.27 | *H* = 2.57, *P* = 0.11, *r* = 0.095 |
| **FTLDCDR-SB**  **(mean±SD)** |  | 8.2±4.1 | 8.7±5.4 | 8.3±5.1 | 9.3±5.7 | *H* = 0.085, *P* = 0.77, *r* = 0.015 | *H* = 0.24, *P* = 0.63, *r* = 0.029 |

Abbreviations: FTLDCDR-SB, Clinical Dementia Rating plus Frontotemporal Lobar Degeneration Behavior and Language Domains sum of boxes; CI, confidence interval; OR, odds ratio; SD, standard deviation


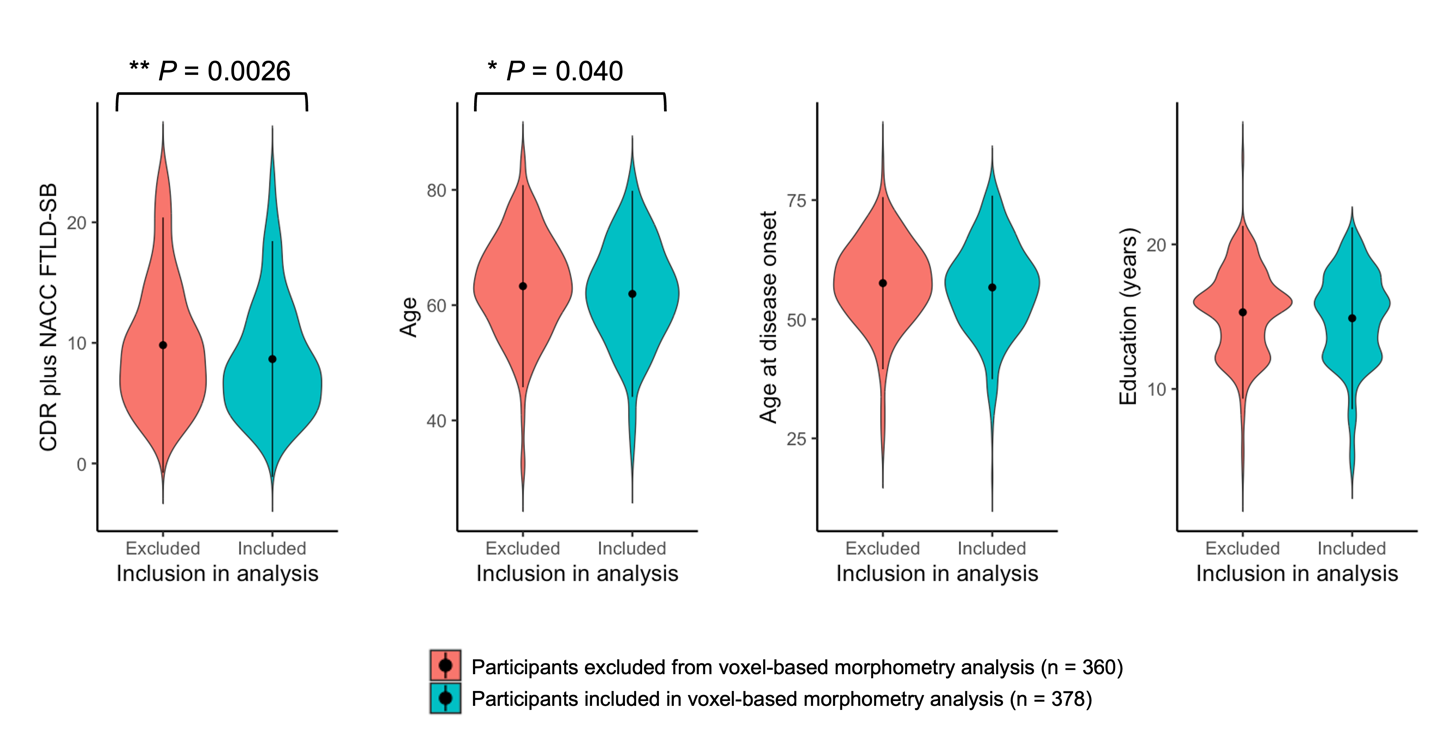


**Supplementary Figure 2**. Characteristics of participants with missing or poor quality T1-weighted MRI scans, which were therefore excluded from analysis (n = 360), and the rest of the participants which were included in voxel-based morphometry analysis (n = 378). These two groups of participants were not significantly different in their age at disease onset (*H* = 2.35, *P* = 0.13, *r* = 0.056) or education years (*H* = 2.01, *P* = 0.16, *r* = 0.052). Participants that were excluded from voxel-based morphometry analysis were more severe in disease (*H* = 9.06, *P* = 0.0026, *r* = 0.11) as assessed by the Clinical Dementia Rating (CDR^®^) Dementia Staging Instrument plus the sum of boxes score of Behavior and Language domains from the National Alzheimer’s Coordinating Center FTLD Module (CDR plus NACC FTLD-SB). Participants that were excluded were also slightly older in age (*H* = 4.34, *P* = 0.040, *r* = 0.077).


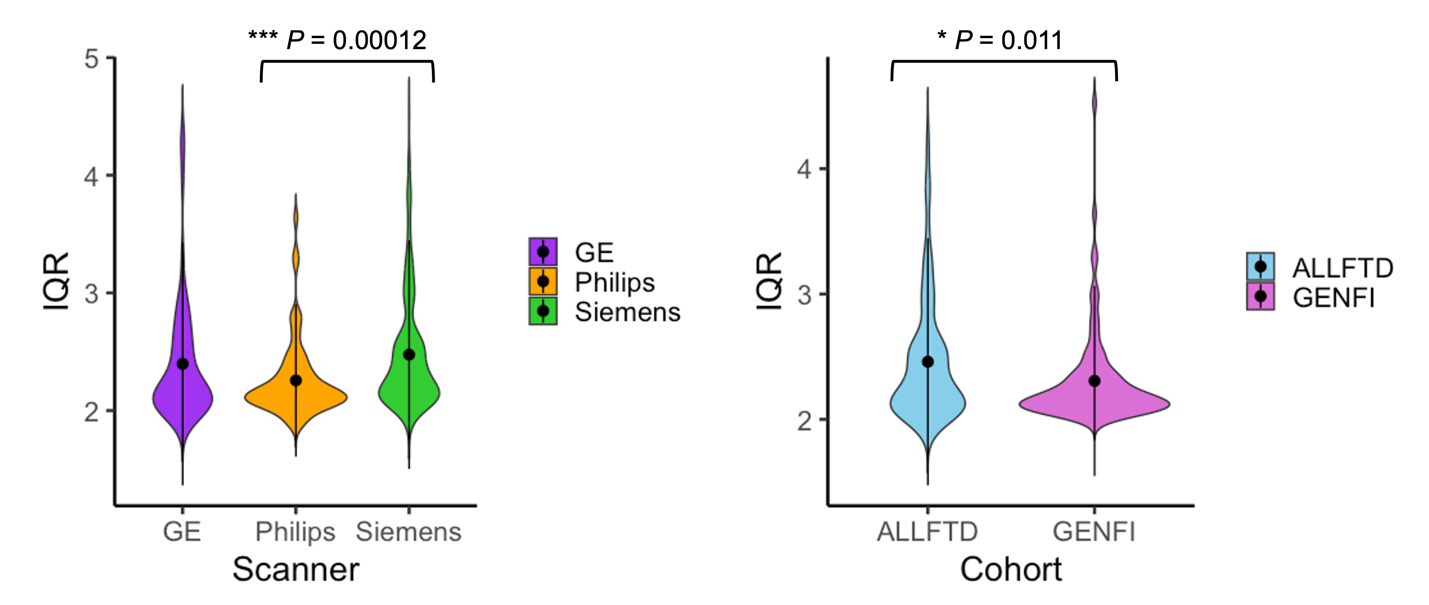


**Supplementary Figure 3.** The weighted overall image quality (IQR) of MRI scans across scanner types and cohorts. The average IQR was significantly different between MRI scans from Philips and Siemens scanners (*H* = 18.62, *P* < 0.001, *r* = 0.22, post-hoc pairwise comparison using the Wilcoxon rank-sum test *P* = 0.00012), and different between MRI scans from the ALLFTD and the GENFI cohorts (*H* = 6.51, *P* = 0.011, *r* = 0.13).


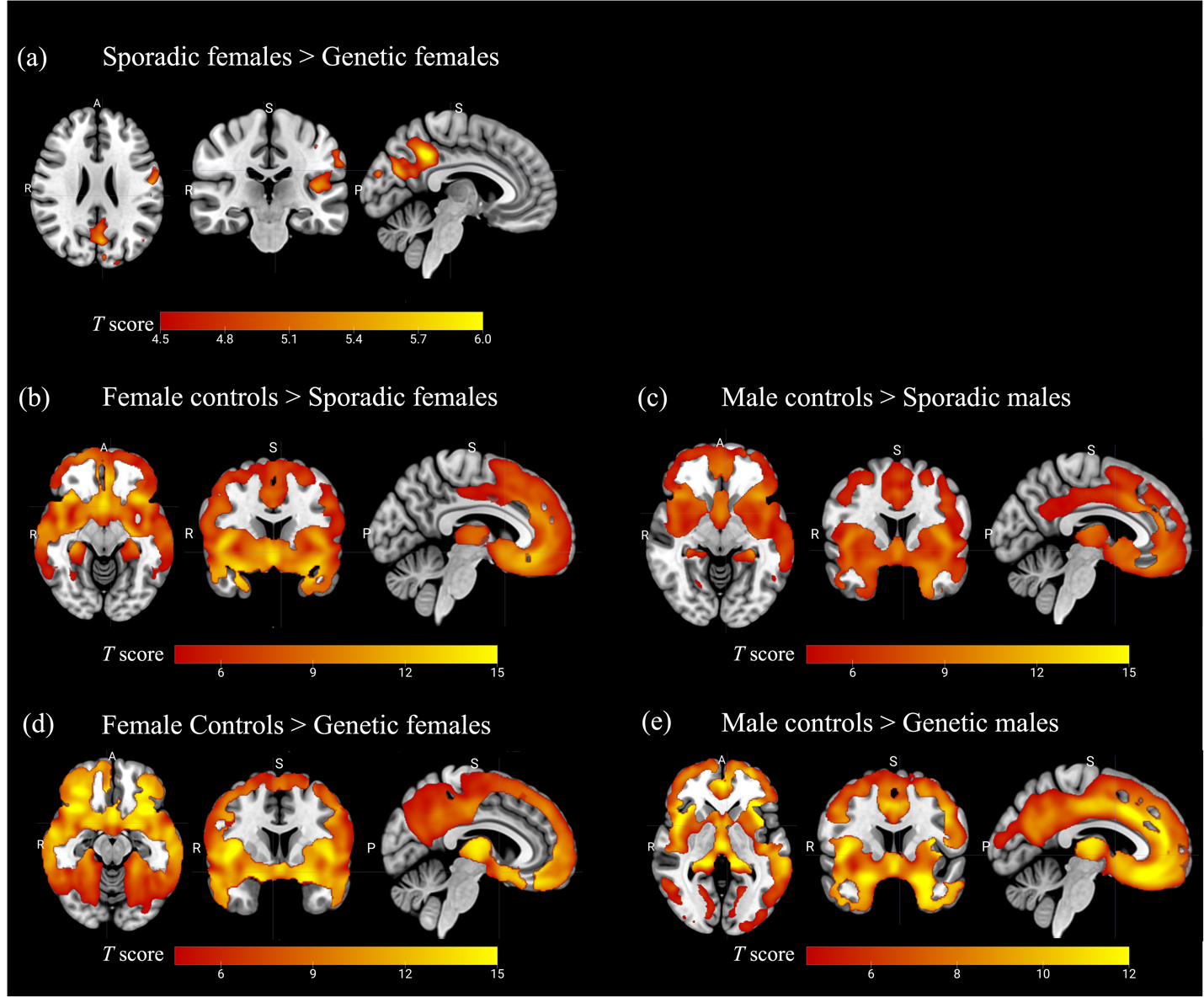


**Supplementary Figure 4.** Voxel-based morphometry analysis results of gray matter volume comparison between a) sporadic bvFTD females and genetic bvFTD females (no significant difference was found in males between sporadic and genetic bvFTD); b) female controls and sporadic bvFTD females; c) male controls and sporadic bvFTD males; d) female controls and genetic bvFTD females; e) male controls and genetic bvFTD males. Results are corrected for age, total intracranial volume, MRI scanner type, and disease severity (disease severity only included in comparison between sporadic and genetic). Results are showing the regions with significant difference (corrected *P* < 0.05).
